# IGD Motifs, Which Are Required for Migration Stimulatory Activity of Fibronectin Type I Modules, Do Not Mediate Binding in Matrix Assembly

**DOI:** 10.1371/journal.pone.0030615

**Published:** 2012-02-15

**Authors:** Lisa M. Maurer, Douglas S. Annis, Deane F. Mosher

**Affiliations:** Departments of Biomolecular Chemistry and Medicine, University of Wisconsin-Madison, Madison, Wisconsin, United States of America; Russian Academy of Sciences, Institute for Biological Instrumentation, Russian Federation

## Abstract

Picomolar concentrations of proteins comprising only the N-terminal 70-kDa region (70K) of fibronectin (FN) stimulate cell migration into collagen gels. The Ile-Gly-Asp (IGD) motifs in four of the nine FN type 1 (FNI) modules in 70K are important for such migratory stimulating activity. The 70K region mediates binding of nanomolar concentrations of intact FN to cell-surface sites where FN is assembled. Using baculovirus, we expressed wildtype 70K and 70K with Ile-to-Ala mutations in ^3^FNI and ^5^FNI; ^7^FNI and ^9^FNI; or ^3^FNI, ^5^FNI, ^7^FNI, and ^9^FNI. Wildtype 70K and 70K with Ile-to-Ala mutations were equally active in binding to assembly sites of FN-null fibroblasts. This finding indicates that IGD motifs do not mediate the interaction between 70K and the cell-surface that is important for FN assembly. Further, FN fragment N-^3^FNIII, which does not stimulate migration, binds to assembly sites on FN-null fibroblast. The Ile-to-Ala mutations had effects on the structure of FNI modules as evidenced by decreases in abilities of 70K with Ile-to-Ala mutations to bind to monoclonal antibody 5C3, which recognizes an epitope in ^9^FNI, or to bind to FUD, a polypeptide based on the F1 adhesin of *Streptococcus pyogenes* that interacts with 70K by the β-zipper mechanism. These results suggest that the picomolar interactions of 70K with cells that stimulate cell migration require different conformations of FNI modules than the nanomolar interactions required for assembly.

## Introduction

**Figure 1:**
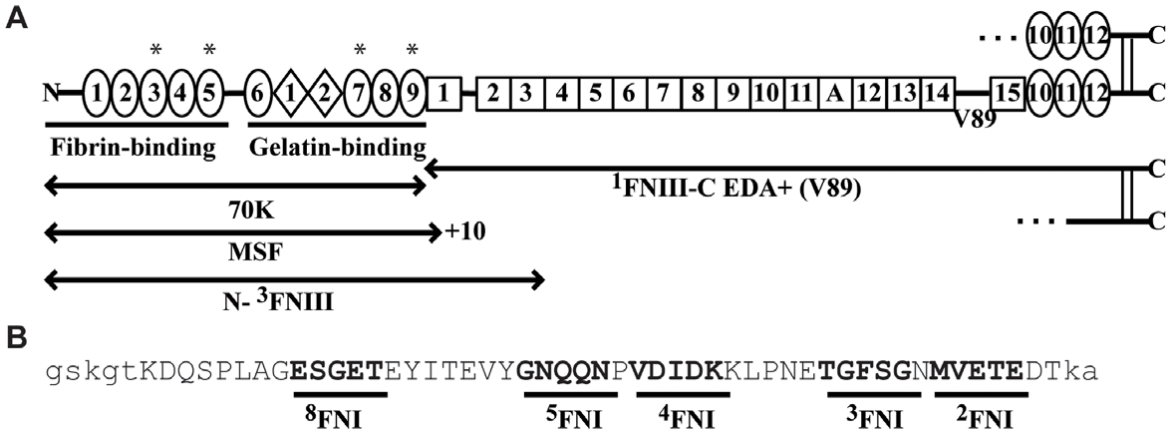
Diagram of FN and FN fragments and location of IGD motifs in 70K. (A) The EDA+, V89 splice variant subunit of FN is shown consisting of 12 FNI modules (ovals), two FNII modules (diamonds), and 16 FNIII modules (squares). Plasma FN lacks EDA and one subunit contains a variable region and the other subunit lacks it. Modules are numbered to facilitate naming recombinant proteins according to modular content. MSF is the N-terminus through the sequence encoded by the ^1^FNIIIa exon and 10 intronic amino acids [6]. FNI modules containing IGD motifs are indicated with an *. (B) Sequence of FUD with presumptive binding sites for FNI modules in bold and underlined and N- and C-terminal tails in lower case.

Fibronectin (FN) is a large glycoprotein distributed widely in the body. It is an insoluble component of the extracellular matrix, where it plays a role in cell adhesion, migration, and embryogenesis [1], [2]. It is also present at near micromolar concentrations in plasma [3], from which it is deposited in fibrin and assembled on the surface of platelets and thus contributes to the growth and stability of thrombi [4], [5]. FN is a disulfide-linked dimer of subunits comprising 12 type 1 (FNI) modules, two type 2 (FNII), and 15–17 type 3 (FNIII) modules [2] (Fig. 1A). Nine of the FNI modules along with the two FNII modules are found in the N-terminal 70-kDa region of FN (70K) (Fig. 1A). This part of FN, as a proteolytic fragment or truncated FN splice variant that also includes part of ^1^FNIII and 10 amino acids encoded by an intron, stimulates cell migration into collagen gels at picomolar concentrations and is called migration-stimulating factor (MSF) (Fig. 1A) [6], [7]. The MSF splice variant of FN is expressed in fetal and cancer fibroblasts and tumor associated macrophages and endothelial cells [6], [8].

FNI modules adopt a characteristic fold with a minor β-sheet (A and B strands), a major β-sheet (C, D, and E strands), two conserved disulfide bonds, and a hydrophobic core containing a conserved tyrosine and tryptophan [9]. The ability of MSF to stimulate fibroblast migration into collagen gels has been attributed to Ile-Gly-Asp (IGD) motifs found in loops between B and C strands of ^3^FNI, ^5^FNI, ^7^FNI, and ^9^FNI [10], [11]. Although it was deduced originally that IGD motifs in ^7^FNI and ^9^FNI are responsible for the migratory effect, N-^5^FNI (Fig. 1A) stimulates fibroblast migration in the presence of vitronectin or serum, indicating that the IGD motifs in ^3^FNI and ^5^FNI are also sufficient for MSF activity [10], [12].

Consecutive FNI modules are arranged “tail-to-head” in a way that allows otherwise unstructured regions of bacterial surface proteins to interact with the E-strands of consecutive modules by anti-parallel β-strand addition, an unusual type of protein-protein interaction that has been termed β-zipper formation [13], [14], [15], [16]. The 70K region also directs binding of FN at nanomolar concentrations to sites on the cell-surface to facilitate FN assembly into insoluble fibrils [17], [18], [19]. In addition, as a fragment, 70K, at nanomolar concentrations, binds to these sites and blocks FN assembly [20]. Remarkably, at the same concentration range that 70K is effective at inhibiting FN assembly, it is inactive in stimulating fibroblast cell migration, *i.e.*, the dose response curve for 70K in the migration assay is bimodal, peaking at ∼15–150 pM and inactive at ∼1.5 nM [7]. We have speculated that β-zipper formation may localize the 70K region of FN to assembly sites based on observations that a 56-residue recombinant polypeptide inspired by the “Functional Upstream Domain” (FUD) of the F1 adhesin in *Streptococcus pyogenes* uses this mechanism to bind to the 70K region of FN with low nanomolar affinity and in so doing blocks FN assembly [14], [21].

Here we compare binding to FN assembly sites and to FUD of wildtype 70K and 70K harboring isoleucine-to-alanine (Ile-to-Ala) mutations in the IGD motifs of ^3^FNI and ^5^FNI, ^7^FNI and ^9^FNI, or ^3^FNI, ^5^FNI, ^7^FNI, and ^9^FNI modules. We conclude that mutations in IGD motifs do not grossly impair the nanomolar affinity interaction between 70K and assembly sites on fibroblasts. In contrast, the Ile-to-Ala mutations alter binding of a monoclonal antibody (mAb) directed towards an epitope in ^9^FNI. These results raise the possibility that mutations of the IGD motifs cause structural changes in FNI modules that impair the picomolar interactions of 70K with the cell components that mediate MSF activity.

## Results

### Expression of 70K with mutations in IGD motif

Previous migration studies focused on the IGD motif of ^7–9^FNI or ^8–9^FNI expressed in *Pichia pastoris* showed that Ile-to-Ala mutations in ^7^FNI and ^9^FNI resulted in loss of MSF activity of the tandem FNI constructs whereas constructs with an intact IGD motif were active [10]. To examine the role of IGD motifs in binding to cell surface sites for FN assembly, we created recombinant 70K proteins in baculovirus containing Ile-to-Ala mutations in the IGD motifs of ^3^FNI and ^5^FNI (70K I150/242A), ^7^FNI and ^9^FNI (70K I480/572A), or ^3^FNI, ^5^FNI, ^7^FNI, and ^9^FNI (70K I150/242/480/572A).

### Mutations in IGD motif do not alter 70K binding

**Figure 2:**
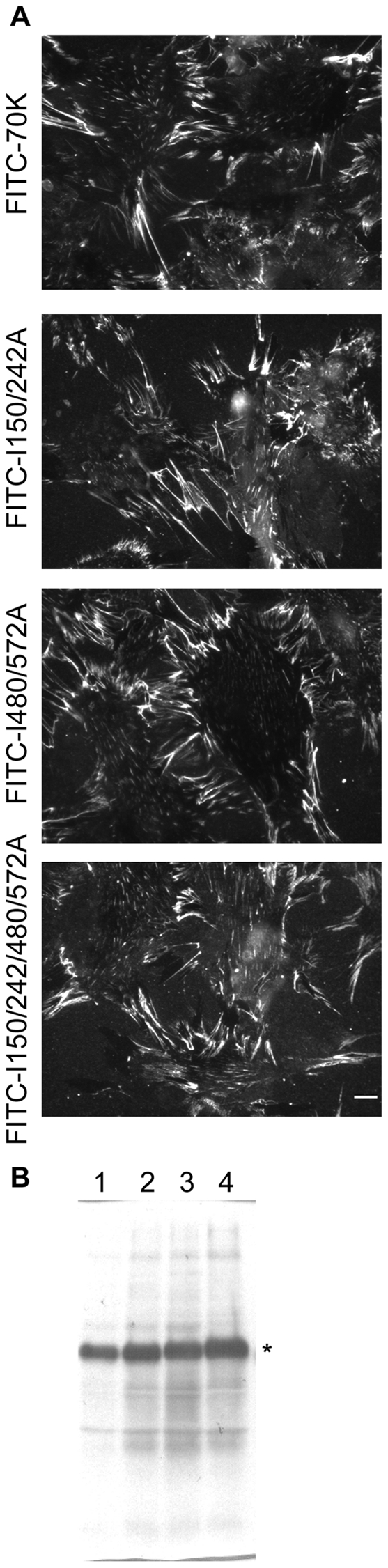
70K with Ile-to-Ala mutations bind to FN^−/−^ fibroblasts. (A) FN^−/−^ fibroblasts adherent to FN-coated coverslips were provided 40 nM FITC-70K, FITC-70K I150/242A, FITC-70K I480/572A, or FITC-70K I150/242/480/572A for 1 h before fluorescent microscopic imaging of the FITC fluorochrome. Results are representative of multiple fields of each condition examined in four separate experiments. Bar, 10 µm. (B) Western blot of cell lysates from cells provided FITC-70K (1), FITC-70K I150/242A (2), FITC-70K I480/572A (3), or FITC-70K I150/242/480/572A (4). The asterisk denotes the FITC-70K band. The Western blot was probed with rabbit anti-FITC followed by peroxidase-conjugated donkey anti-rabbit IgG.

To determine the effect of mutations in IGD motifs on 70K binding to FN-null (FN^−/−^) fibroblasts, cells adherent to adsorbed FN were provided 40 nM FITC-70K, FITC-70K I150/242A, FITC-70K I480/572A, or FITC-70K I150/242/480/572A, a concentration of 70K that saturates ∼80% of binding sites on the cell surface [22]. All proteins bound to the surface of FN^−/−^ fibroblast in fibrillar arrays as seen by microscopy (Fig. 2A). Western blotting corroborated the impression from microscopy that similar quantities of the proteins bound to the cell layers (Fig. 2B). These results indicate that intact IGD motifs are not required for binding of 70K to cell-surface assembly sites.

### N-^3^FNIII binds to the cell-surface

**Figure 3:**
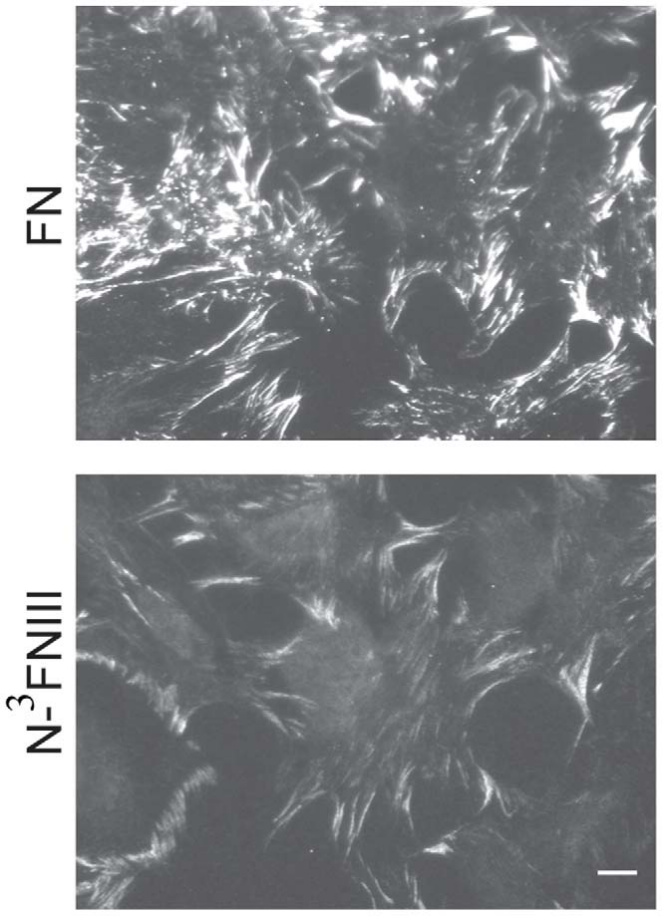
N-^3^FNIII binds to FN^−/−^ fibroblasts. FN^−/−^ fibroblasts adherent to ^1^FNIII-C EDA+-coated coverslips were provided 30 nM N-^3^FNIII or FN subunit (15 nM dimeric FN) in the presence of 200 nM LPA for 3 h, washed, fixed, and immunostained with the mAb 5C3 followed by rhodamine labeled secondary antibody. Bar, 10 µm.

Previous studies have shown that 70K, but not N-^3^FNIII, stimulates fibroblast migration [7]. The inability of N-^3^FNIII to stimulate fibroblast migration has been attributed to a salt bridge between Arg222 in ^4^FNI and residues in ^3^FNIII that causes N-^3^FNIII to fold back on itself, thus occluding IGD motifs in ^7^FNI and ^9^FNI [7]. To look for other differences between binding to cell surface assembly sites and MSF activity, we tested the ability of N-^3^FNIII to bind FN^−/−^ fibroblasts. FN^−/−^ fibroblasts adherent to ^1^FNIII-C EDA+ coated coverslips were provided 30 nM N-^3^FNIII or FN (molarity based on the subunit) and stimulated with 200 nM lysophosphatidic acid. After 3 h, cells were fixed and stained with mouse-anti human antibody 5C3, which recognizes ^9^FNI [14]. As visualized by 5C3 staining, N-^3^FNIII or FN bound to FN^−/−^ fibroblasts (Fig. 3).

### Alteration of the epitope of mAb 5C3

**Figure 4:**
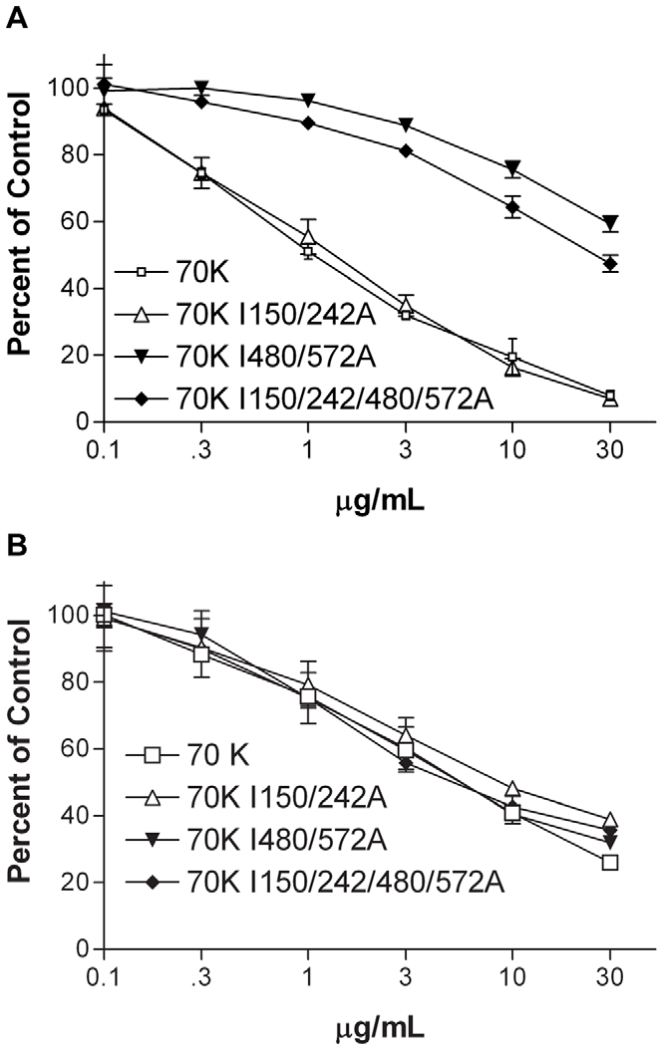
Structural alterations in 70K with IGD mutations. Binding relative to no competitor of 1∶30,000 5C3 ascites (A) or 1∶50,000 7D5 ascites (B) to coated FN in the presence of increasing concentration of 70K (□), 70K I150/242A (▵), 70K I480/572A (▾), or 70K I150/242/480/572A (♦). Values are mean plus/minus standard deviation of 3 experiments.

Previous NMR studies comparing ^8–9^FNI structures with any of seven different mutations in the IGD motif in ^9^FNI showed minor changes in residues surrounding the mutations, and no changes in the folds of the modules [10]. As another probe of structural alterations, we used mAb 5C3, which binds Gly567 in the loop between the A and B strands, five residues away from Ile572 in the IGD motif [14]. In a competitive ELISA, increasing concentrations of 70K or 70K I150/242A decreased binding of 5C3 to coated FN; 30-fold higher concentrations of 70K I480/572A or 70K I150/242/480/572A were needed for the same effect (Fig. 4A). In contrast, when we tested mAb 7D5 to an epitope in the loop adjacent to the E-strand of ^4^FNI [14], 70K, 70K I150/242A, 70K I480/572A, or 70K I150/242/480/572A decreased antibody binding to coated FN at approximately equal concentrations (Fig. 4B). Thus, the I572A mutation causes structural changes that are significant enough to alter the affinity of 5C3 that binds in close proximity to the site of the mutation.

### IGD mutations minimally alter bacterial peptide binding

**Figure 5:**
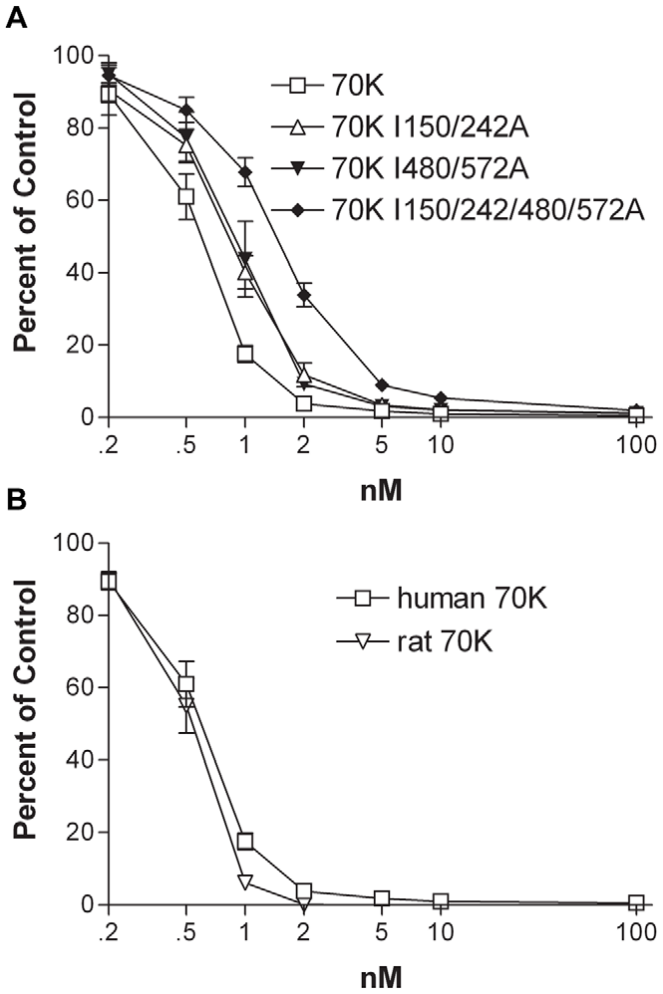
70K with Ile-to-Ala mutations compete less well for binding of b-FUD to adsorbed FN. (A) Binding relative to no competitor of 0.3 nM b-FUD to coated FN in the presence of increasing concentrations of 70K (□), 70K I150/242A (▵), 70K I480/572A (▾), or 70K I150/242/480/572A (♦). (B) Binding relative to no competitor of 0.3 nM b-FUD to FN in the presence of increasing concentrations of human 70K (□) or rat 70K (▿). Values are mean plus/minus standard deviation of 3 experiments.

The functional upstream domain is a 49-amino acid sequence from the F1 adhesin in *S. pyogenes* that binds to 70K by β-strand addition and inhibits FN matrix assembly [14], [19], [21]. To determine if Ile-to-Ala mutations altered binding of FUD to 70K, we looked at the ability of soluble 70K and mutant 70Ks to compete for binding of 0.3 nM biotinylated FUD (b-FUD) to coated FN. Both I150/242A and 70K I480/572A had 2-fold decreased ability to compete for binding of b-FUD to coated FN as compared to wildtype 70K (Fig. 5A). 70K with all four Ile-to-Ala mutations had an even greater decrease in its ability to compete for b-FUD (Fig. 5A). To determine if the decreased affinity of b-FUD for mutated 70K was specific for Ile-to-Ala mutations, we compared the ability of human and rat 70K to compete for b-FUD binding to FN. There are 30 residues that differ in the expressed rat 70K protein as compared to the human protein [14]. However, increasing concentrations of human 70K or rat 70K had the same ability to compete for 0.3 nM b-FUD binding to coated FN (Fig. 5B). Further, human 70K in which Asn-Gly-Arg (NGR) motifs in ^3^FNI and ^5^FNI were mutated to Gln-Gly-Arg (QGR) sequences [23] competed equally well as 70K for b-FUD binding to coated FN (data not shown).

## Discussion

The 70K fragment of FN binds to cell-surface FN assembly sites with the same nanomolar affinity as full-length FN, and at a thousand-fold lower picomolar concentration, 70K stimulates migration of fibroblasts into type I collagen [7], [22]. Full-length FN and the N-^3^FNIII construct lack MSF activity, probably because the sites required for the activity are obscured in the quaternary structure [7], [12]. The structure/function relations of the MSF activity compared to FN assembly activity are not known. Because migration stimulating ability of 70K has been shown to be dependent on the IGD motifs [10], [11], we investigated binding of wildtype 70K and 70K with Ile-to-Ala mutations in ^3^FNI and ^5^FNI; ^7^FNI and ^9^FNI; or ^3^FNI, ^5^FNI, ^7^FNI, and ^9^FNI to the cell surface as well as to mAbs and FUD polypeptide that bind to 70K [14].

Ile-to-Ala mutations in either two or four IGD motifs in 70K did not affect the binding of 70K to FN^−/−^ fibroblasts and had minimal effects on binding of FUD. These results contrast with loss of MSF activity when isoleucine residues in the IGD motifs of ^7^FNI and ^9^FNI were mutated in a FN fragment containing ^7–9^FNI [10]. The contrasting results indicate that the interactions with cell surfaces that stimulate migration and enable FN assembly are different. Additional evidence supporting the idea that the interactions are different is provided by experiments showing that N-^3^FNIII binds to the cell-surface of FN^−/−^ fibroblasts, whereas N-^3^FNIII does not stimulate migration [7]. Finally, a polypeptide highly homologous to FUD has been demonstrated to enhance rather than inhibit the ability of N-^3^FNIII to stimulate migration whereas FUD is an inhibitor of binding of 70K to fibroblast assembly sites [19], [24].

Signaling mediating MSF activity seems to involve inhibition of AKT [25]. The cell-surface molecules that initiate such signaling remain to be identified. Experiments blocking αvβ3 integrins with antibodies indicate a role for this integrin in migration [11], [12], but whether αvβ3 interacts with MSF to initiate signaling or engages binding sites in the supporting collagen gel to mediate migration is not known. We are not aware of αvβ3 interacting with any ligands with picomolar affinity. In addition, there is no evidence that the nanomolar binding of 70K to the cell surface assembly sites is dependent on αvβ3. A cyclic RGD peptide that inhibits αvβ3 cell adhesive activity does not block binding of 70K at nanomolar concentrations to the cell-surface [23]. Further, although NGR motifs in ^3^FNI and ^5^FNI spontaneously convert to integrin-binding *iso*DGR sequences, and *iso*DGR can interact with αvβ3 integrins [26], the conversion of NGR to *iso*DGR is incomplete, and mutagenesis experiments indicate that the sequences are not responsible for binding of 70K to assembly sites [23].

Previous NMR studies showed little structural alteration in ^9^FNI with mutations in the IGD motif; these changes involved Ser575 and the disulfide connecting the A- and D-strands [10]. Our results showed that the I572A mutation results in decreased binding of mAb 5C3 to its epitope, which contains Gly567 five amino acids away from Ile572. Further, mutations in the IGD motif, but not the 30 differences between rat and human 70K or mutations in NGR motifs, affected the ability of 70K to interact with the bacterial peptide FUD. As with other FN-binding sequences from bacteria [13], [15], [16], FUD appears to bind to the E-strand of FNI modules [14]. Because the IGD sequence is in the loop connecting B and C strands, it is unlikely that mutations in IGD motifs interfere directly with FUD binding [12]. Instead, because the conserved isoleucine is part of the hydrophobic core of FNI modules [9], we hypothesize that the Ile-to-Ala mutations disrupt the hydrophobic core of the FNI module which in turn deforms the modules and alters the ability of FUD to bind. The IGD motif is part of the sequence YX(I/V)G(D/E) found in nine of 12 FNI modules. It is noteworthy that mutation of the tyrosine, which is found in all 12 FNI modules and contributes to the hydrophobic core[9], to serine in any of the five N-terminal FNI modules is deleterious to secretion of 70K and binding of 70K to matrix assembly sites or to *Staphylococcus aureus* [27], an interaction that requires β-zipper formation.

The literature and present results present a conundrum. The interaction leading to migration is not only orders of magnitude tighter than described integrin-ligand interactions, but also orders of magnitude tighter than the interaction of 70K to FUD or assembly sites. Nevertheless, the MSF interaction apparently involves recognition of a binding surface that can be presented by any of four different FNI modules whereas the binding to assembly sites or FUD involves simultaneous interactions with multiple FNI modules. The attribute required for MSF activity may be the display of the side chains of the IGD motifs on the surface of the modules, as suggested by the MSF activity of micromolar concentrations of IGD-containing tetrapeptides [11], or a common structural feature of IGD-containing FNI modules that requires the isoleucine.

## Materials and Methods

### Expression of recombinant proteins, purification of FN, and antibodies

Recombinant human 70K (N-^9^FNI) with I150/242A, I480/572A, or I150/242/480/572A mutations and rat 70K were produced using a baculovirus expression system with either High 5 or SF9 cells as described before [14], [28]. Proteins were purified over nickel-nitrilotriacetic acid agarose as previously described except that in the case of the 70K proteins, proteins were incubated with nickel-nitrilotriacetic acid agarose in the presence of 15 mM imidazole [28], [29]. Residues in 70K proteins are numbered starting with the initiating methionine of FN. Thus, the residue that we call Ile572 would be called Ile541 if numbering were to start with the first residue after removal of FN's pre-pro sequence [7]. 70K proteins were stored in 10 mM Tris, 300 mM NaCl, 1 M NaBr. Recombinant N-^3^FNIII was expressed and purified as described previously [14]. ^1^FNIII-C EDA+ was described previously [29]. Expression of FUD was described previously [14]. The sequence of FUD is shown in Fig. 1B.

Human plasma FN was prepared by anion exchange chromatography of a fibrinogen-rich fraction as described previously [30]. Mouse anti-human mAbs 7D5 and 5C3 were described previously [14].

### Labeling of 70K and FUD

70K was labeled with fluorescein-5-isothiocyanate (FITC) (Invitrogen) as previously described [23]. FUD was biotinylated with N-hydroxysulfosuccinimide-biotin (Pierce) as previously described [14].

### 70K binding

FN^−/−^ mouse fibroblasts were derived from stem cells of FN knockout mice as previously described [14], [29]. For assembly experiments, coverslips coated with 10 µg/mL FN (20 nM) or 3.8 µg/mL (10 nM) ^1^FNIII-C EDA+ were provided 70,000 cells in 0.5 mL Dulbecco's modified Eagle's medium (DMEM, Invitrogen) plus 0.2% bovine serum albumin (BSA) as described previously except that cells were allowed to spread for 1 h before the addition of 40 nM FITC-70K proteins for 1 h [14]. In experiments comparing the deposition of N-^3^FNIII or FN, cells adherent to ^1^FNIII-C EDA+ were provided 30 nM N-^3^FNIII or 30 nM FN (monomer, 15 nM dimer) with 200 nM lysophosphatidic acid (LPA) for 3 h. Cells were fixed and immunostained with 2 µg/mL 5C3 followed by rhodamine red-x donkey anti-mouse IgG (Jackson ImmunoResearch). For Western blot, cells were treated as above except that cells were grown in 24-well tissue culture treated plates (Costar 3524) coated with 10 µg/mL FN. Instead of fixation, cells were provided 40 µL SDS-PAGE sample buffer containing 10% β-mercaptoethanol, incubated for 10 min, and sample buffer was collected after forceful scraping. This was repeated with 10 µl of sample buffer also containing 10% β-mercaptoethanol. Samples were run on an 8% SDS-PAGE gels and Western blot was done with rabbit anti-FITC (Molecular Probes) and peroxidase-conjugated donkey anti-rabbit IgG (Jackson ImmunoResearch) according to standard procedure [29], [31].

### Enzyme-linked assay

Enzyme-linked assays were done using high binding plates (Corning 3590) coated overnight at 4°C with 50 µl of 10 µg/mL FN. Assays were preformed as previously described [14]. Graphs are of mean +/− standard deviation of the mean of triplicates from 3 separate experiments.
